# Use of Parental Disability Trajectories to Identify Adolescents Who are Young Carers

**DOI:** 10.1007/s10964-022-01627-z

**Published:** 2022-05-20

**Authors:** Tania L. King, Marissa Shields, Martin O’Flaherty, Anne Kavanagh, Matthew J. Spittal

**Affiliations:** 1 Disability and Health Unit, Centre for Health Equity, Melbourne School of Population and Global Health, The University of Melbourne, Carlton, VIC Australia; 2 Life Course Centre, Institute for Social Science Research, The University of Queensland, Brisbane, QLD Australia; 3 Centre for Mental Health, Melbourne School of Population and Global Health, The University of Melbourne, Carlton, VIC Australia

**Keywords:** Disability, Disadvantage, Caring, Young carer, Adolescence

## Abstract

Being a young carer can have significant impacts on the lives of children and adolescents. Identifying young carers is difficult, making the provision of support challenging for service providers. This sample contained 4464 Australian children/adolescents across 11 years (49% female, aged 6/7 years at baseline, and 16/17 years at final wave). Group-based trajectory modeling was applied to examine parental disability trajectories across 5 waves of data collection. Associations between estimated trajectories and unpaid/informal caring at age 16/17 years were then assessed. Three trajectory groups were identified: *consistently-low* (80%), *low-increasing-high* (10%) and *moderate*-*high* (10%) levels of parental disability. There was strong evidence that caring was elevated in the *low-increasing-high* group compared to the *consistently-low* group, and moderate evidence of elevation in the *moderate*-*high* group. By identifying adolescents with increased odds of becoming young carers, this study shows that parental disability may be an important way for service providers to identify and support young carers.

## Introduction

Young carers are children, adolescents and young adults who provide regular or significant unpaid care to a family member with a disability, chronic health condition, substance dependency or who is otherwise in need of regular care and assistance (Becker, 2007; Leu & Becker, 2019). Young carers are one of the most invisible, under-recognized and under-supported groups in society (Becker, 2007; Kavanaugh et al., 2016; Wong, 2017). Much of this lack of recognition is due to the challenges of identifying young carers, yet identifying young carers is key to ensuring that they receive the support and services needed. Finding characteristics or factors that predict young caring therefore represents a key research need and may assist in better identification of young carers. In this study, this need is addressed by investigating parental disability trajectories of Australian children over the period 2006–2014 (when children were aged 6/7–14/15 years) to assess the extent to which these trajectories are associated with young caring at age 16/17 years.

Internationally, the prevalence of young carers in industrialized countries is estimated to be between 2–8% of young people aged under 18 years (Joseph et al., 2020), although estimates vary depending on definitions and methodology. In Australia (Cass et al., 2009), as in other countries such as the UK (Dearden et al., 2004), parents are commonly the recipients of the care provided by young carers. ﻿In Australia in 2018, 18% of the population were living with a disability, however the prevalence increased substantially with age: between the ages of 5–44 years, the prevalence of disability remained relatively static, and after 44 years, the prevalence increased across age groups (ABS, 2019). The age at which the prevalence of disability starts to increase aligns with the period when many adults are parenting adolescents; this means that for a substantial proportion of adolescents, their parents will develop a health condition or disability that requires assistance. A proportion of these adolescents become young carers.

Enumerating and identifying young carers is difficult for a number of reasons; often young carers do not consider themselves to be carers, and therefore do not engage with formal services (Smyth et al., 2011). Researchers have also claimed that under-identification can be attributed to a lack of awareness among some service providers and agencies, a failure on behalf of potential gatekeepers such as teachers and health professionals to identify them, and stigma related to the disability or condition of the person they are caring for (Cass et al., 2011). Under-identification is also sometimes due to an unwillingness on the part of young carers to self-identify due to fear of adverse intervention, such as removal of the young person (or the person being cared for) into other care arrangements (Cass et al., 2011).

### Theoretical Frameworks: Life-course Theory and Social Determinants of Health

The principal conceptual and theoretical frameworks underpinning this research are life-course theory (Elder et al., 2003; Graham & Power, 2004) and the social determinants of health framework (SDOH) (Marmot et al., 2008). Both life-course and SDOH theories acknowledge that the future lives of young carers are molded and shaped by a complex set of influences at different levels – community, societal, family, peer and cultural. Life course theory considers how human lives are shaped by experiences, events and exposures from birth to death, and recognizes the importance of the timing (in terms of life-stage, life transitions, chronological age) and time (in terms of historical and cultural context, social change) of these events (Elder et al., 2003). It recognizes adolescence as a crucial developmental period in which many behaviors are established that are carried through to adulthood (Graham & Power, 2004). The SDOH framework (Marmot et al., 2008) recognizes that people’s life outcomes are influenced by conditions (social determinants) that are largely modifiable. These include the conditions in which people are born, grow, work, live and age, as well as the broader forces and systems that shape these conditions, such as social policies, social norms, and political systems (Marmot et al., 2008). The experiences of caring in childhood and adolescence can shape the future lives of young carers. Education, for example, is an important social determinant of health (Marmot et al., 2008): if the education of young carers is curtailed due to their caring role, this may influence future employment and consequent economic prospects across the life-course. This in turn may affect the opportunities afforded to young carers, as well as the conditions in which they live and work, and all of these factors may influence their physical and mental health outcomes. Applying the life-course approach and the SDOH does not necessarily mean that the aim is to prevent caring; instead, it recognizes the potency of these early experiences in shaping lives and highlights the importance of acting to mitigate any potentially damaging effects.

### Effects of Young Caring

Caring can have enduring effects on the lives of those providing it, including young carers. Studies examining the outcomes of young caring have shown that it impacts on the education of young carers (Cass et al., 2009; Stamatopoulos, 2018), with many young carers unable to complete homework or engage in educational offerings due to caring responsibilities (Becker & Sempik, 2018). The social and recreational activities of young carers are also frequently constrained (Cass et al., 2009), often precluding the establishment and consolidation of social networks at a life stage when this is of vital importance. There is also evidence that the health and wellbeing of young carers is impacted by their caring responsibilities, with effects particularly observed on mental health outcomes (King et al., 2021; Tseliou et al., 2018). In a sample of 246 children aged 8–18 years whose parents had a severe physical or mental illness or substance abuse, almost half reported stress, and negative outcomes at a level to be of clinical concern were noted in 10% of research participants (Kallander et al., 2018). A census based record linkage study found that while young carers were less likely than their peers to report chronic mobility problems, they were more likely to have chronic poor mental health, and had higher mortality risk than their peers (Tseliou et al., 2018). Using augmented inverse probability weighting on a sample of 2165 Australian adolescents, it was found that caring at age 14/15 years was associated with poorer mental health four years later (King et al., 2021). However not all effects of caring are negative. Recent evidence indicates that young caring is associated with benefit finding (the extent to which one can perceive benefits following adversity) (Wepf et al., 2021a), and among young carers, benefit finding was associated with better mental health (Wepf et al., 2021b).

### Parental Disability: What is Known About its Relationship with Young Caring

Acknowledging that caring is not always deleterious for young carers, young carers do need to have support systems available to them. To enable such support, identifying young carers and understanding the factors and characteristics that lead to young caring is critical. The presence of a family member with a disability or health condition is one key characteristic that is associated with young caring. Among a community sample of 2474 Australian children, cross-sectional analysis showed that the presence of a family member with a disability or serious medical condition was associated with more intensive caregiving, and these effects were particularly pronounced among those caring for parents (Pakenham & Cox, 2015). In a sample of 135 young people aged 10–24 who lived with a family member who was ill or disabled, having a mother who was in need of care (rather than a father or sibling) was associated with the provision of more socio/emotional care (keeping company, supporting emotional needs), instrumental support (paying bills, shopping, arranging appointments) and household/domestic tasks (Ireland & Pakenham, 2010). The adoption of caring roles among children whose parents have a disability or health condition has been observed across many studies, including qualitative studies of children whose parents have: multiple sclerosis (Turpin et al., 2008); amyotrophic lateral sclerosis/motor neuron disease (Kavanaugh et al., 2021); or varied impairments or disabilities (Bolas et al., 2007).

### Limitations in Current Knowledge

Of the work that has been conducted among young carers, much has been qualitative. While critical in informing understanding of the experiences of young carers, the relative paucity of quantitative studies means that there is a lack of evidence quantifying the effects of caring, as well as a lack of evidence about the causal factors leading to young caring. Understanding of the typical pathways that may precede caring is also limited by the fact that most previous quantitative studies of young carers, particularly in relation to parental disability or health condition, have used cross-sectional data. It is also the case that in some countries, a lack of large-scale national data limits young carer scholarship, and overall, there is a lack of longitudinal studies (Kavanaugh et al., 2016). Longitudinal data, where the temporal ordering of events is apparent, is needed to unpack associations between parental disability and young caring. Additionally, most previous quantitative studies have used samples of specific population groups, for example young carers, or children of parents with disability, limiting understanding of the extent to which young caring is occurring at the population level, and also how to identify young carers within the broader population. This highlights the need for studies using large population representative samples to advance understanding of how to identify young carers within society.

Given that most young carers are caring for a parent with a disability or physical or mental health condition, assessing and understanding the typical trajectories of parental disability/health condition is important. There are likely to be households in which a parent has a disability or health condition from when the child was born, while for many households, no parent will experience a disability. There may also be households in which parents experience intermittent disabilities, and others still where a disability or health condition is acquired by a parent – either early in a child’s life, or later when the child is an adolescent. There may be other common trajectories of parental disability and examining these trajectories could enable better understanding of the provision of care for parents with disabilities. Further, examining the way these trajectories are associated with young caring is vital and could also inform the development of appropriate supports for young carers.

It is also the case that parental disability may be experienced differently and at different timepoints by groups of young people, and this will likely have implications on the provision of caring. Some young people may have parents who have a disability or health condition throughout their lives. Families in such circumstances may be more likely to have access to, or set up, the resources and supports needed early on in a way that does not require the child/adolescent to provide care, while households in which parents acquire a disability later may not readily access supports. Such households may become more reliant on the child/adolescent (who at the point of parental disability acquisition may be older and more capable of helping). Alternatively, it may be the case that in households in which a parent experiences a sustained disability, or where there is intermittent disability, there is greater reliance on the assistance of children from early in the child’s life. Recognizing this variability and examining different trajectories in relation to young caring could assist in identifying young people most likely to become a young carer, as well as avenues to support these young carers.

## Current Study

The overall lack of research on young carers using large longitudinal datasets, as well as the need to understand how trajectories of parental disability may be associated with young caring represent key research gaps that this study aimed to address. Using a large representative sample of children/adolescents, this study sought to identify parental disability trajectories over time and examine the way these trajectories relate to young caring. Using 6 waves of data, the specific objectives of this research were to: describe trajectories in parental disabilities across 5 timepoints (from the age of 6/7 years to 14/15 years); examine the characteristics of adolescents following different parental disability trajectories; and assess differences in odds of caring at age 16/17 years among adolescents whose parents experience different disability trajectories. It was hypothesized that distinct trajectories of parental disability would emerge for adolescents (however there were no expectations about the number or shape of trajectories) (Hypothesis 1) and that if trajectories emerged that showed sustained disability among parents, the children/adolescents of these parents would have greater odds of reporting caring at Wave 7 (Hypothesis 2).

## Methods

This study adhered to Strengthening the Reporting of Observational Studies in Epidemiology (STROBE) statement. Group-based trajectory modeling (GBTM) methods were used to identify trajectories of parental disability and assess these in relation to young caring. To do this, six waves of data were used from the Longitudinal Study of Australian Children (LSAC), a nationally representative longitudinal study of Australian children and their families.

### Cohort and Study Design

LSAC commenced in 2004 among two cohorts: families with 4–5-year old children (Cohort K) and families with 0–1-year old children (Cohort B), and has been collected biennially since (Soloff et al., 2005). A two-stage clustered sampling approach was carried out, with 311 postcodes selected, and then children residing in these postcodes randomly selected from the Medicare Australia database (which has near complete coverage of Australian residents) (Soloff et al., 2005).

Due to the availability of variables and the study focus on adolescent outcomes, this analysis used Waves-2-7 of Cohort K (age 6/7-years to 16/17-years). While there has been strong retention across the waves, response rates have declined from 89.6% of the original sample in Wave-2, to 62.0% in Wave-7 (Bandara et al., 2020). The choice of waves was determined by two key factors: (1) the timepoints at which key measures were collected; (2) a strategy to maximize the use of as much data as possible across the dataset. To assess trajectories of parental disability, it was desirable to use as many waves as possible, hence this was assessed from Waves 2-6. Informal caring was assessed in LSAC among adolescents at Waves 6 (age 14/15 years), 7 (16/17 years), 8 (18/19 years). While many young people aged over 18 years provide informal care, it is also the case that 18 years marks a common transition from schooling into tertiary education or employment, and it is at this time that many young people move out of home. The research focus was on young people aged under 18 years, and for this reason, this study used the caring measure from Wave 7 (age 16/17 years of age).

### Variables

#### Parental disability

We used measures of parental disability collected at Waves-2-6 for parent 1 (child’s biological mother for 96% of Wave-2 respondents) and parent 2 (in 93% of two parent households, this was the child’s biological father). Parents were asked “Does [Parent 1 or 2] have any medical conditions or disabilities that have lasted, or are likely to last, for 6 months or more?” Prompt cards with a range of conditions, including mental illness, were presented. Responses for both parents were combined and dichotomized, with response categories: no parent in the household has a disability; one or more parents have a disability.

#### Caring status

A binary variable was used to assess informal caring status. As the focus of this study was on informal (unpaid) caring by adolescents, rather than adults, the caring variable that was assessed in Wave-7 (adolescent age 16/17-years) was used. Caring was based on the following question: “Do you help someone who has a long-term health condition, has a disability or is elderly with activities that they would have trouble doing on their own?”. Valid responses were “yes” or “no”, and respondents were instructed to think about help given, or that they are likely to give, for at least six months. They were also asked not to include help given as part of a paid job, unpaid volunteer work or community service.

#### Covariates

Covariates identified as potential confounders of the association between parental disability trajectories and caring outcomes were collected in Wave 2. These included: gender (male, female); parents in household (single parent, two parent household); household income (quintiled); parental country of birth (both parents born in Australia, one or more parents born elsewhere); First Nations identity (one or more parent identifies as Indigenous, non-Indigenous); maternal education (did not complete Year 12, Year 12, certificate/trade, diploma, bachelor degree or higher); maternal age at Wave 2 (under 30 years, 30–34 years, 35–39 years, 40–44 years, 45 years and over); and number of siblings in household (continuous).

### Statistical Analyses

#### Group based trajectory modeling

GBTM is a type of finite mixture modeling used to identify groups of individuals who follow similar trajectories on an outcome variable (Nagin, 2015). GBTM was used to classify patterns in parental disability (presence of a parent with disability in the study child’s house). In GBTM, the distribution of the outcome (in this case parental disability), conditional on time or age, is the fundamental concept of interest (Jones & Nagin, 2013). The trajectories represent the average development or progression of the outcome over time (van der Nest et al., 2020), and the groups that arise from GBTM are conceived as discrete groups that may be present within the population (Nagin, 2015). The GBTM approach principally seeks to identify latent longitudinal strata ﻿comprising individuals who are following a similar course across time (Nagin, 2014), however it may also assist in identifying the timing of transitions among different groups. Applying a multinomial modeling strategy, GBTM uses maximum likelihood estimation (Jones & Nagin, 2013) meaning that the coefficients remain unbiased in the presence of missing data, assuming such data are missing at random (Nagin & Odgers, 2010).

The *traj* user-written command was used (Jones & Nagin, 2013, 2012), in Stata SE Version 16 (StataCorp, 2019), specifying the binary logit distribution and modeled parental disability as a function of time, with no predictors. ﻿The mean time between waves ranged from 23.2 months (waves 2–3) to 25.0 months (waves 1–2), with variance increasing across waves from 3.8 (waves 1–2) to 11.7 (waves 6–7). Survey sample weights were included in models (Usback et al., 2018), thereby allowing calculation of the population prevalence of parents with a disability at each time point with standard errors that respect LSAC’s sampling strategy.

﻿The group based trajectory modeling used here was conducted in concordance with the Guidelines for Reporting on Latent Trajectory Studies (GRoLTS) checklist (van de Schoot et al., 2017) (see supplementary material Table S1). Nagin’s approach to identifying the optimal model was followed (Nagin, 2015). A series of 1-5-group models were tested to identify the number of latent trajectories in the model and specified different polynomial functions to determine the shape of each trajectory. Refer to Tables S6, S7 in the supplementary material for details of this process. After identifying the optimal trajectory model, individuals were then assigned to trajectory groups based on their maximum posterior probability of group membership.

#### Missing and non-response

Of the 4983 children participating in LSAC Cohort K in Wave 1, 519 (10.4%) did not participate in any further waves of data collection and were therefore excluded from analyses. This left a total of 4464 participants included in the trajectory analysis, all of whom had complete parental disability data at Wave 2. Comparison of the sample with those who were missing from the trajectory analysis (did not participate in Waves 2–7 of LSAC) indicated that those missing did not differ from the sample in terms of gender (see Tables S3–S5 in supplementary material for details of non-response and missingness). However, a higher proportion of those who were missing (only participated in Wave 1) were: from a single parent household; had at least one parent from a non-English speaking country; and had a mother who had not completed Year 12 (high school).

#### Multinomial and logistic regression analyses

In the second stage of analysis, multinomial logistic regression modeling was used to characterize children/adolescents following different parental disability trajectories, according to baseline demographic characteristics. Associations between estimated parental disability trajectories and caring in Wave 7 (age 16/17 years) were then assessed using logistic regression models.

Given data were missing on several key covariates (household income, mothers education, parental country of birth and parental indigenous status) as well as the caring variable, multiple imputation (MI) using chained equations with 100 imputations was carried out. The above noted covariates and the caring variable were included in the imputation model, as well as the following ancillary variables: presence of two parents in household, study child gender, area remoteness, birth plurality, relationship of parent figure to child. Baseline characteristics of the trajectory groups are also based on the imputed sample.

The main multinomial and logistic regression analyses were carried out using multiple imputation methods. To ensure that the multiple imputation methods had not distorted results, sensitivity analysis that used complete case data with sample weights was undertaken (Tables S8, S9 in supplementary material).

## Results

### Baseline Characteristics

Wave 2 (baseline) sample characteristics using multiple imputation methods are presented in Table 1. Most respondents lived in two parent households, and for over two thirds of respondents, both parents were born in Australia. Just under half of respondents had one other sibling living in the household, and approximately one third lived in a household in the middle household income quintile. There was good Indigenous representation in the sample, with over 5% of respondents having one or more parents who identified as Indigenous. At Wave 2, over one tenth of the sample had at least one parent with a disability.

**Table 1:** Wave 2 sample characteristics (*n* = 4464)^a^

Variable	Category	%(95%CI)
Gender	Male	51.0(49.5, 52.5)
	Female	49.0(47.5, 50.5)
Parents in household	Two parents	85.2(84.2, 86.3)
	Single parent	14.8(13.7, 15.8)
Highest education of mother	Did not complete Year 12	18.5(17.4, 19.7)
	Year 12	13.3(12.3, 14.3)
	Certificate/trade	29.8(28.4, 31.1)
	Diploma	8.8(8.0, 9.6)
	Bachelor degree or higher	29.6(28.3, 31.0)
Parental country of birth	Both parents born in Australia	68.2(66.8, 69.6)
	1+ parent born in English-speaking	15.4(14.3, 16.5)
	1+ parents born in non-English speaking country outside Australia	16.4(15.3, 17.5)
First nations identity	Non-indigenous	93.7(92.5, 94.8)
	Indigenous	6.3(5.2, 7.5)
Household income (quintile)	1-lowest	11.2(10.2, 12.1)
	2	21.1(19.8, 22.3)
	3	32.4(31.0, 33.8)
	4	23.0(21.7, 24.2)
	5-highest	12.4(11.4, 13.3)
Number of siblings in household	0	9.1(8.3, 10.0)
	1	45.2(43.7, 46.7)
	2	30.6(29.2, 31.9)
	3	15.1(14.1, 16.2)
Parental disability	No parent with a disability	88.3(87.4, 89.3)
	One or more parents with a disability	11.7(10.7, 12.6)
Maternal age	<30 years	8.3(7.5, 9.1)
	30–34 years	22.8(21.6, 24.0)
	35–39 years	38.2(36.7, 39.6)
	40–44 years	23.0(21.8, 24.3)
	45 years and over	7.7(6.9, 8.5)
Caring status^b^ (Wave 7)	No caring	80.1(78.5, 81.6)
	Any caring	19.9(18.4, 21.5)

This table presents the sample characteristics, measured at Wave 2.

^a^Proportions are calculated following multiple imputation.

^b^Caring status measured in Wave 7, all other variables presented here measured in Wave 2

### Trajectory Analysis

**Fig. 1:**
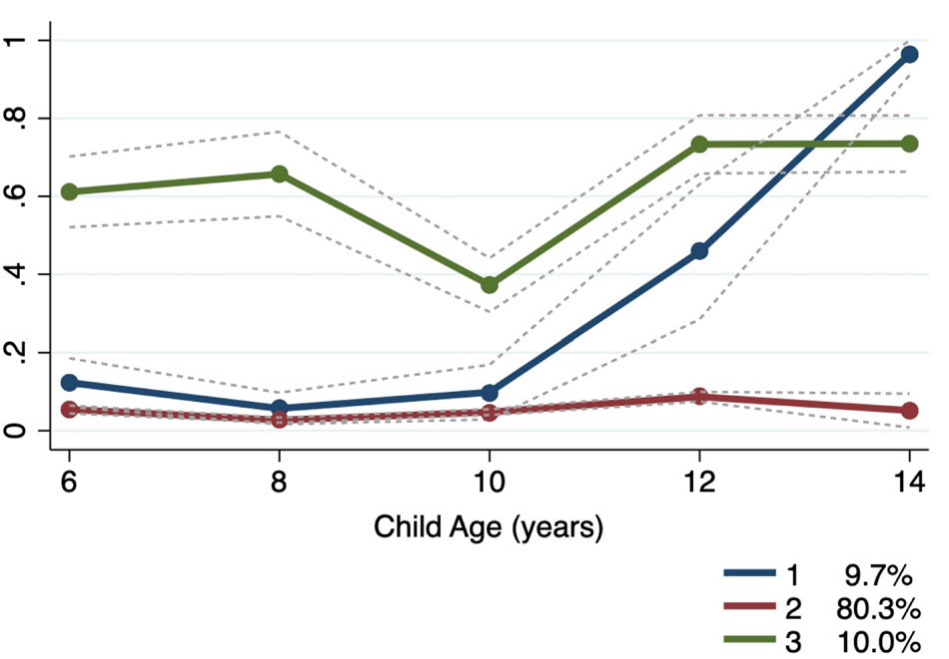
Estimated trajectories and observed group means for parental disability from ages 6 to 14 years (Waves 2–6). This figure plots the estimated mean trajectories for the optimal 3-group model

The optimal model was identified as a 3-group model, with a quadratic, cubic and quartic polynomial component respectively (see Table S7 in supplementary material) and is presented in Fig. 1. Trajectory groups were categorized as *consistently*-*low*, *low-increasing-high*, and *moderate*-*high* probability of parental disability. Most respondents (80.3%) were classified in the *consistently-low* trajectory group, where the presence of parental disability was low or not present, with high stability over the course of data collection. Of the remaining sample, 10% were categorized in the *moderate-high* group, where the probability of parental disability was relatively constant and moderately high, and the remaining 9.7% were in the *low-increasing-high* group. The *low-increasing-high* group mirrored the trajectory of the *consistently-low* group for Waves 2–4 (child ages 6–10 years), after which there was an increase in the probability of parental disability for Waves 5 and 6 (child/adolescent ages 12–14 years). For each group the mean posterior probability of group membership and the odds of correct classification respectively were 0.95 and 4.6 (*consistently-low*), 0.61 and 14.5 (*low-increasing-high*), 0.80 and 37.6 (*moderate-high*). Further details on the trajectory analysis methodology are presented in the supplementary material.

In adjusted multinomial analyses, the relative risk ratios (RRRs) for membership in each trajectory group according to Wave 2 characteristics were derived (Table 2). The RRR is the ratio of the relative risk (for membership in each group compared to the *consistently-low* group) for each unit change in the predictor (Wave 2) variable. There was evidence that having a mother who did not complete year 12, at least one parent born in an English-speaking other country, maternal age (having a mother aged 40–44 or 45 years or older at Wave 2) and being in the two lowest quintiles for household income increased likelihood of membership in the *moderate-high* trajectory (compared to the *consistently-low* group). Having any number of siblings and living in a single parent household was associated with a reduced likelihood of being in the *moderate-high* group compared to the *consistently-low* group. ﻿Having a mother aged 45 years or more at Wave 2 was the only sociodemographic characteristic that predicted likelihood of being in the *low-increasing-high* group relative to the *consistently-low* group.

**Table 2:** Results of multinomial logistic regression model: relative risk ratios for trajectory group membership according to Wave 2 demographic variables (*n* = 4464)

Variable	Trajectory group				
	*Consistently-low* *n* = *3609*	*Low-increasing-high* *n* = *452*		*Moderate-high* *n* = 403	
	RRR (95%CI)	RRR (95%CI)	*p*-value	RRR (95%CI)	*p*-value
*Gender*					
Male	Ref	Ref	Ref	Ref	Ref
Female	Ref	0.94 (0.77, 1.15)	*p* = 0.548	0.84 (0.68, 1.03)	*p* = 0.099
*Household type*					
2 parent household	Ref	Ref	Ref	Ref	Ref
Single parent household	Ref	0.81 (0.57, 1.16)	*p* = 0.252	0.53 (0.37, 0.77)	*p* = 0.001
*Mother’s education*					
Bachelor	Ref	Ref	Ref	Ref	Ref
Did not complete Year 12	Ref	0.92 (0.67, 1.27)	*p* = 0.622	1.47 (1.06, 2.05)	*p* = 0.022
Year 12	Ref	1.12 (0.81, 1.55)	*p* = 0.486	1.23 (0.84, 1.80)	*p* = 0.278
Certificate	Ref	0.83 (0.63, 1.10)	*p* = 0.198	1.28 (0.95, 1.73)	*p* = 0.104
Diploma	Ref	1.05 (0.72, 1.51)	*p* = 0.815	1.09 (0.71, 1.67)	*p* = 0.710
*Parental country of birth*					
Both born Australia	Ref	Ref	Ref	Ref	Ref
1+ parent born English speaking other	Ref	0.92 (0.69, 1.23)	*p* = 0.569	1.35 (1.02, 1.79)	*p* = 0.036
1+ parent born non-English speaking other	Ref	0.79 (0.59, 1.05)	*p* = 0.105	0.71 (0.52, 0.98)	*p* = 0.035
*First Nations identity*	Ref				
1+ parent Indigenous	Ref	0.85 (0.48, 1.51)	*p* = 0.583	0.91 (0.51, 1.62)	*p* = 0.755
No parent Indigenous	Ref	Ref	Ref	Ref	Ref
*Household income (quintile)*	Ref				
1-lowest	Ref	1.17 (0.72, 1.88)	*p* = 0.529	4.01 (2.40, 6.69)	*p* < 0.001
2	Ref	1.16 (0.80, 1.68)	*p* = 0.447	2.70 (1.72, 4.24)	*p* < 0.001
3	Ref	0.95 (0.67, 1.33)	*p* = 0.754	1.53 (0.99, 2.36)	*p* = 0.056
4	Ref	0.91 (0.64, 1.28)	*p* = 0.578	1.32 (0.84, 2.07)	*p* = 0.224
5-highest	Ref	Ref	Ref	Ref	Ref
*Number of siblings in house*	Ref				
0	Ref	Ref	Ref	Ref	Ref
1	Ref	0.80 (0.56, 1.14)	*p* = 0.216	0.61 (0.44, 0.86)	*p* = 0.005
2	Ref	0.81 (0.56, 1.18)	*p* = 0.279	0.53 (0.37, 0.76)	*p* = 0.001
3	Ref	0.90 (0.60, 1.36)	*p* = 0.631	0.66 (0.44, 0.98)	*p* = 0.039
*Maternal age*					
<30 years	Ref	Ref	Ref	Ref	Ref
30–34 years	Ref	1.46 (0.95, 2.25)	*p* = 0.086	1.34 (0.86, 2.09)	*p* = 0.200
35–39 years	Ref	1.22 (0.80, 1.87)	*p* = 0.357	1.23 (0.80, 1.89)	*p* = 0.352
40–44 years	Ref	1.37 (0.88, 2.15)	*p* = 0.163	2.06 (1.33, 3.20)	*p* = 0.001
45 years and over	Ref	1.89 (1.14, 3.14)	*p* = 0.014	2.39 (1.44, 3.96)	*p* = 0.001

This presents the relative risk ratios for membership in each trajectory group according to Wave 2 characteristics. The relative risk ratio is calculated, relative to the *consistently-low* group, for each unit change in the Wave 2 variable.

### Informal Caring in Wave 7

Table 3 presents the proportion of the sample reporting that they provided informal care at Wave 7 by trajectory group. Most of the sample carried out no informal caring, with less than one quarter of each trajectory group reporting any caring at Wave 7. The proportion of caring was lower among those in the *consistently-low* group, but there was little difference between the *low-increasing-high* group, and the *moderate-high* group.

**Table 3:** Reported caring activity in sample at Wave 7 (age 16/17 years, *n* = 4464)^a^

		Trajectory group		
		Consistently-low *n* = 3609	Low-increasing-high *n* = 452	Moderate-high *n* = 403
Any caring (Wave 7)	No caring	81.1(79.4, 82.8)	75.9(71.7, 80.2)	75.7(70.8, 80.6)
	Caring	18.9(17.2, 20.6)	24.1(19.8, 28.3)	24.3(19.4, 29.2)

This table presents the overall proportion of adolescents reporting caregiving activity at the age of 16/17 years, measured at Wave 7

^a^Proportions are calculated following multiple imputation

In logistic regression models, the odds of caring at Wave 7 for trajectory groups were estimated. Table 4 shows that there was little difference between adjusted and unadjusted models, and so reporting focuses on the adjusted results. Compared to the *consistently-low* group, the *low-increasing-high* group had increased odds of caring at Wave 7 (OR 1.35, 95%CI 1.04–1.73). Those in the *moderate*-*high* group also appeared to have increased odds of caring at Wave 7 (OR 1.30, 95%CI 0.97–1.73) compared to the *consistently-low* group, however this association was not statistically significant. Results of sensitivity analysis conducted on the complete case sample (*n* = 2398, see Table S9 in Supplementary material) did not substantively differ from main findings.

**Table 4:** Odds ratios for caring at age 16/17 years (Wave 7) by parental disability trajectory group (imputed sample, *n* = 4464)

		Consistently-low *n* = 3609	Low-increasing-high *n* = 452	Moderate-high *n* = 403
Caring (W7, age 16/17 years)	Unadjusted	Ref	1.36 (1.06, 1.75)	1.38 (1.04, 1.82)
	Adjusted^a^	Ref	1.35 (1.04, 1.73)	1.30 (0.97, 1.73)

This table presents the unadjusted and adjusted results of logistic regression analyses assessing the relationship between parental disability trajectory group and caring at age 16/17 years.

^a^Models adjusted for gender, parents in household, maternal education, maternal age, household income, number of siblings in household, parental country of birth, First Nations identity

## Discussion

Across many societies, young carers make substantial contributions to the provision of care. While there are positive effects of caring, such as strong family bonds, a sense of responsibility, pride and maturity, greater acceptance of others (Cass et al., 2009), it can also have ongoing adverse effects on the lives of those providing it. For service providers, identifying young carers is challenging, and this means that young carers often do not receive the support that they need. Within research on young carers, there has been an overall lack of quantitative research deploying longitudinal methods to identify factors or characteristics that are associated with young caring. This study sought to address this gap and provide evidence to guide service providers in the delivery of services to young carers. It aimed to use a large, population representative dataset to quantitatively examine trends in parental disability over time and assess this in relation to young caring.

In this research three distinct trajectories of parental disability or health condition were identified (supporting Hypothesis 1). These trajectories were: *consistently-low, low-increasing-high, moderate-high*. In alignment with Hypothesis 2, it was found that, relative to the *consistently-low* group, individuals in the *low-increasing-high* and *moderate*-*high* parental disability groups had increased odds of being a young carer at the age of 16/17 years. This is the first study that the authors are aware of to examine how parental disability, assessed across several years in a child’s early life, is associated with young caring in adolescence. A substantial number of studies have documented young caring by drawing on samples of children whose parents have disabilities or health conditions (Bolas et al., 2007; Ireland & Pakenham, 2010; Kavanaugh et al., 2021; Turpin et al., 2008). Furthermore, cross-sectional studies have shown associations between parental disability and young caring (Pakenham & Cox, 2015), but no studies have used longitudinal data to document trajectories in parental disability and assess the associations between these trajectories and young caring utilizing a study design that specifies the temporal ordering in which disability trajectories precede young caring.

There were no baseline household characteristics that distinguished the *consistently-low* group from the *low-increasing-high* group except for maternal age. These results may suggest that the *low-increasing-high* group is representative of those adolescents whose parents have unexpectedly (or after a sustained period of good health) experienced disability or a health condition, perhaps related to parental ageing, over the course of the study period. By contrast, some key characteristics are associated with increased likelihood of being categorized in the *moderate*-*high* group, including low maternal education, maternal age, living in a low-income household and having at least one parent born in an English-speaking country outside Australia. This aligns with evidence that people with disability in Australia have lower education and are more likely to be living in low income households (Kavanagh et al., 2013), and it is likely that parents in this group had experienced their disability or ill health over a sustained period, with associated and ongoing socioeconomic disadvantage in their living circumstances. The fact that having siblings was associated with reduced likelihood of being in the *moderate-high* group may be related to age of parent and sibling order. In this sample, having a mother aged 40-44 or 45 years or older at Wave 2 (when the child was 6/7 years of age) was associated with membership in the *moderate-high* trajectory, and it is known the prevalence of disability increases with age (ABS, 2019). In relation to siblings, it is possible that those children/adolescents in the *moderate-high* group had siblings older than 15 years (and therefore not included as siblings in this measure). It is also possible that for some parents, having a chronic health condition or disability limited the number of children they had. It is unclear why living in a single parent household at baseline may be associated with a reduced likelihood of being in the *moderate-high* group. It is possible that this pertains to the fact that having two parents in the household provides two chances that a parent will have a disability, however countering this, an additional parent would also potentially reduce the need for children/adolescents to take on a caring role.

The fact that being in the *low-increasing-high* and *moderate-high* parental disability groups increased the odds of being a young carer at the age of 16/17 years comports with other young carer literature showing that parental disability may precipitate the assumption of a caring role among young people (Aldridge & Becker, 1999), and that many children and adolescents who have a parent with a disability or health condition provide care for them (Ireland & Pakenham, 2010; Pakenham & Cox, 2015). Young caring can impact many domains of life, including mental health and wellbeing (King et al., 2021; Tseliou et al., 2018), education (Becker and Sempik, 2018), and recreational activities (Cass et al., 2009). However it is important to acknowledge that not all caring experiences are negative for young people (Aldridge, 2006; Fives et al., 2013). Among some young carers, positive effects of caring have been observed, including strengthening of the relationship between parents and young carers (Aldridge, 2006). Furthermore, not all children whose parents have a disability provide care; research has shown that some parents with disability actively seek to shield their children from caring responsibilities (Prilleltensky, 2004). The objective then, is not to position parental disability or health conditions as problematic, but to identify ways to appropriately identify and support young people who may be caring for parents with disability, and in doing so mitigate potential negative impacts.

### Implications of These Results

Quantitative young carer scholarship is an underdeveloped area of research, and this study makes an important contribution to the evidence base. By pinpointing groups of young people who have greater odds of being a young carer, this work contributes evidence to inform efforts to identify young carers. Specifically, these results show that children or adolescents whose parents are known to have a sustained health condition or disability or acquire an injury/disability, are more likely to be providing informal care, and may therefore be in need of support themselves. ﻿Identifying young carers is difficult for a number of reasons; often young carers do not consider themselves to be carers, and therefore do not engage with formal services (Smyth et al., 2011). Under-identification can also be attributed to a lack of awareness among some service providers and agencies, and a failure on behalf of potential gatekeepers to identify them (Cass et al., 2011). The demand for care is increasing across societies worldwide, with increasing pressure being placed on informal carers (Deloitte Access Economics, 2015), including young carers. Given this, the need to find ways to identify young carers is all the more critical.

It is also important to acknowledge that adolescence is a key formative stage when a confluence of developmental processes – biological, cognitive, social - mark the transition to adulthood (Sawyer et al., 2018; Viner et al., 2012). The experiences and exposures occurring during this period are foundational in influencing future health and wellbeing (Sawyer et al., 2018). It is also important to note that the context of adolescence is shifting, with large-scale transformations across societies, including: greater, and increasing rates of participation in education; increasing urbanization; emerging global labor markets (and associated with this, new forms of work), and changes in relationship and household formation (Woodman & Wyn, 2015). In most developed countries, the transition into adult roles and responsibilities is occurring later than for previous generations, with most young people experiencing a prolonged adolescence (Sawyer et al., 2018). This is not true of young carers however - many of whom are adopting adult roles significantly earlier than their peers – and marks them as even more different to their peers.

By highlighting the association between parental disability trajectories and young caring, this work shows that parental disability may represent an important way to identify young carers. Identifying young carers is the first step in improving young carers lives, however understanding the support they need, whether educational support, care respite, emotional support or counseling, and then delivering such support, is the next step. Recent evidence suggests that to effectively support young carers, there is a need for integration and cooperation of services across sectors (Nap et al., 2020). This requires whole system approaches that bring services together in a unified way, rather than operating in isolation of the broader family context. For example, services providing support to a parent with a disability/health condition should be integrated with other sectors such as education to ensure that teachers and schools can provide educational support to young carers.

### Strengths and Limitations

There are some key strengths and limitations of this analysis. Regarding limitations: first, the GBTM approach characterizes trajectories as continuous distributions that represent distinct groups and cannot account for within group heterogeneity. It is also true that when examining the trajectories as exposures in relation to an outcome, in this case caring, adjusting for confounders is not straightforward (Song, 2019). This modeling adjusted for confounders collected in Wave 2, however this does not acknowledge the time-varying nature of many confounders, and the direction of the bias introduced by potential time-varying confounders is unclear. The caring measure used in this analysis was self-reported and may be subject to measurement error. Furthermore, the dichotomous nature of the caring measure does not distinguish between frequent, intensive caring, and more infrequent or intermittent caring. Finally, it is likely that the measure of parental disability used in this analysis captured a substantial level of heterogeneity. There is likely to be significant variation in the severity and type disability, and the extent to which the disability/health condition required care. Further to this, this measure of disability does not distinguish between those households in which one or two parents had a disability. This research, however, takes the perspective of the child. Accordingly, the issue then is whether any parent, as opposed to how many, has a disability or limiting health condition. Furthermore, the caring measure used here does not define who is being cared for, thus it is not known whether those who are caring for someone are caring for a parent or someone else. It is noted however, that the point of this analysis was to elucidate ways for service providers to identify young carers. Service providers will not necessarily know the composition of a household nor other details– this analysis shows however, that parental disability provides them with a means of identifying children/adolescents who have greater odds of being a young carer.

In terms of strengths, rigorous methods and a large dataset representative of the Australian population of young people were used. ﻿With a wide and rich set of covariates, this dataset provides strong statistical power and enabled us to identify distinct group trajectories as well as characteristics associated with them. Weighting was applied, and multiple imputation was carried out to derive an imputed analytic sample to minimize bias due to sample attrition and non-representativeness (noting that while response rates for LSAC are generally high, there were some missing data due mainly to loss to follow-up). The main results arising from multiple imputation were corroborated by sensitivity analysis using complete case data. Caring was self-reported by study adolescents and parental disability was reported by household informants (parents); thus, for the logistic regression analysis of associations between trajectories and odds of caring, the risk of exposure-outcome dependent measurement error was minimized. While there is an element of subjectivity in the selection of the best-fitting GBTM model, best practice methods were used and the approach adopted was clearly documented in the application of the GRoLTs checklist (van de Schoot et al., 2017).

## Conclusion

Informal caring can have substantial impacts on the lives of young carers. In recognition of the gap in quantitative longitudinal research among young carers, as well as limited understanding of how parental disability over time is associated with young caring, this study sought to examine trajectories in parental disability over multiple timepoints, and then assess these trajectories in relation to young caring. Three distinct trajectories of parental disability/health condition were identified across five waves of data. Most respondents lived with parents with low probability of disability across the five time points (*consistently-low)*. For one group of respondents there was a stable and moderately high probability of parental disability or health condition sustained across the five time points (*moderate-high*). For a similar proportion of respondents, there was a low probability of disability/health condition initially, with increasing probability over latter waves suggesting the onset of disability occurred during the data collection period (*low-increasing-high*). The *low-increasing-high* and the *moderate-high groups* had greater odds of providing care at the age of 16/17 years, relative to those respondents in the *consistently-low* group. These results have implications for programs and services, as they provide evidence that adolescents whose parents have experienced sustained disability, or who have acquired a disability are more likely to be providing informal care to their parents. Children and adolescents whose parents have a disability may need support, given their greater odds of becoming a young carer, and this evidence should inform the delivery of integrated services to better support young carers.

## Supplementary Information

Supplementary Material

## References

[CR1] ABS (2019). *Disability, Ageing and Carers, Australia: Summary of Findings*. https://www.abs.gov.au/statistics/health/disability/disability-ageing-and-carers-australia-summary-findings/latest-release

[CR2] Aldridge J (2006). The experiences of children living with and caring for parents with mental illness. Child Abuse Review.

[CR3] Aldridge J, Becker S (1999). Children as carers: the impact of parental illness and disability on children’s caring roles. Journal of Family Therapy.

[CR4] Bandara, D., Gasser, C., Jessup, K., Renda, J., Warren, D., & Daraganova, G. (2020). *Factors associated with non ‑ response in Growing Up in Australia: The Longitudinal Study of Australian Children*. *LSAC technical paper* No.23 (Issue 23).

[CR5] Becker S (2007). Global perspectives on children’s unpaid caregiving in the family. Global Social Policy.

[CR6] Becker, S., & Sempik, J. (2018). Young adult carers: The impact of caring on health and education. *Children & Society*, chso.12310. 10.1111/chso.12310

[CR7] Bolas H, Van Wersch A, Flynn D (2007). The well-being of young people who care for a dependent relative: An interpretative phenomenological analysis. Psychology and Health.

[CR8] Cass, B., Brennan, D., Thomson, C., Hill, T., Purcal, C., Hamilton, M., & Adamson, E. (2011). *Young carers: Social policy impacts of the caring responsibilities*. https://www.sprc.unsw.edu.au/media/SPRCFile/1_Young_Carers_Report_Final_2011.pdf

[CR9] Cass, B., Smith, C., Hill, T., Blaxland, M., & Hamilton, M. (2009). Young Carers in Australia:Understanding the Advantages and Disadvantages of Their Care Giving. In *SSRN Electronic Journal* (Issue 38). 10.2139/ssrn.1703262

[CR10] Dearden, C., Becker, S., & Carers, Y. (2004). *Young Carers in the UK: the 2004 report*. https://www.lboro.ac.uk/microsites/socialsciences/ycrg/youngCarersDownload/YCReport2004%5B1%5D.pdf

[CR11] Deloitte Access Economics. (2015). *The economic value of informal care in Australia in 2015*. www.deloitte.com/au/about

[CR12] Elder, G. H., Kirkpatrick Johnson, M., & Crosnoe, R. (2003). The Emergence and Development of Life Course Theory. In J. T. Mortimer & M. J. Shanahan (Eds.), *Handbook of the Life Course**:* (pp. 3–19). Springer.

[CR13] Fives A, Kennan D, Canavan J, Brady B (2013). Critical Social Work Why we still need the term “Young Carer”: Findings from an exploratory study of young carers in Ireland. Critical Social Work.

[CR14] Graham, H., & Power, C. (2004). *Childhood disadvantage and adult health: a lifecourse framework*. London: Health Development Agency. 10.1111/j.1365-2214.2004.00457.x 15527477

[CR15] Ireland MJ, Pakenham KI (2010). The nature of youth care tasks in families experiencing chronic illness/disability: Development of the Youth Activities of Caregiving Scale (YACS). Psychology and Health.

[CR16] Jones B, Nagin D (2013). A note on a stata plugin for estimating group-based trajectory models. Sociological Methods and Research.

[CR17] Jones, B., & Nagin, D. S. (2012). *A STATA Plugin for estimating group-based trajectory models*. Heinz College Research.

[CR18] Joseph S, Sempik J, Leu A, Becker S (2020). Young carers research, practice and policy: An overview and critical perspective on possible future directions. Adolescent Research Review.

[CR19] Kallander EK, Weimand B, Ruud T, Becker S, Van Roy B, Hanssen-Bauer K (2018). Outcomes for children who care for a parent with a severe illness or substance abuse. Child and Youth Services.

[CR20] Kavanagh AM, Krnjacki L, Beer A, Lamontagne AD, Bentley R (2013). Time trends in socio-economic inequalities for women and men with disabilities in Australia: Evidence of persisting inequalities. International Journal for Equity in Health.

[CR21] Kavanaugh MS, Henning F, Mochan A (2021). Young carers and ALS/MND: exploratory data from families in South Africa. Vulnerable Children and Youth Studies.

[CR22] Kavanaugh MS, Stamatopoulos V, Cohen D, Zhang L (2016). Unacknowledged Caregivers: A Scoping Review of Research on Caregiving Youth in the United States. Adolescent Research Review.

[CR23] King T, Singh A, Disney G (2021). Associations between young informal caring and mental health: a prospective observational study using augmented inverse probability weighting. The Lancet Regional Health - Western Pacific.

[CR24] Leu, A., & Becker, S. (2019). Young Carers. In H. Montgomery (Ed.), *Oxford bibliographies in childhood studies*. Oxford University Press.

[CR25] Marmot M, Friel S, Bell R, Houweling TA, Taylor S (2008). Closing the gap in a generation: health equity through action on the social determinants of health. The Lancet.

[CR26] Nagin D (2014). Group-based trajectory modeling: An overview. Annals of Nutrition and Metabolism.

[CR27] Nagin, D. (2015). Group-Based Modeling of Development. In *Group-Based Modeling of Development*. Harvard University Press. 10.4159/9780674041318

[CR28] Nagin D, Odgers C (2010). Group-based trajectory modeling in clinical research. Annual Review of Clinical Psychology.

[CR29] Nap, H. H., Hoefman, R., Jong, N. de, Lovink, L., Glimmerveen, L., Lewis, F., Santini, S., D’Amen, B., Socci, M., Boccaletti, L., Casu, G., Manattini, A., Brolin, R., Sirk, K., Hlebec, V., Rakar, T., Hudobivnik, T., Leu, A., Berger, F., … Hanson, E. (2020). The awareness, visibility and support for young carers across Europe: a Delphi study. *BMC Health Services Research*, 1–16. 10.21203/rs.3.rs-22136/v1 10.1186/s12913-020-05780-8 PMC7540437 33028311

[CR30] Pakenham KI, Cox S (2015). The effects of parental illness and other ill family members on youth caregiving experiences. Psychology and Health.

[CR31] Prilleltensky O (2004). My child is not my carer: Mothers with physical disabilities and the well-being of children. Disability and Society.

[CR32] Sawyer SM, Azzopardi PS, Wickremarathne D, Patton GC (2018). The age of adolescence. The Lancet Child & Adolescent Health.

[CR33] Smyth C, Blaxland M, Cass B (2011). So that’s how I found out I was a young carer. Journal of Youth Studies.

[CR34] Soloff, C., Lawrence, D., & Johnstone, R. (2005). *Longitudinal study of Australian children technical paper no*. *1: Sample design*. https://growingupinaustralia.gov.au/sites/default/files/tp1.pdf

[CR35] Song M (2019). Trajectory analysis in obesity epidemiology: A promising life course approach. Current Opinion in Endocrine and Metabolic Research.

[CR36] Stamatopoulos V (2018). The young carer penalty: Exploring the costs of caregiving among a sample of Canadian youth. Child & Youth Services.

[CR37] StataCorp. (2019). *Stata Statistical Software: Release 16*. StataCorp LLC.

[CR38] Tseliou F, Rosato M, Maguire A, Wright D, O’Reilly D (2018). Variation of Caregiver Health and Mortality Risks by Age. American Journal of Epidemiology.

[CR39] Turpin M, Leech C, Hackenberg L (2008). Living with Parental Multiple Sclerosis: Children’s Experiences and Clinical Implications. Canadian Journal of Occupational Therapy.

[CR40] Usback, S., Bureau, A., Household, S., & Methodology, S. (2018). *Wave 7 weighting and non-response* (Issue 20).

[CR41] van de Schoot R, Sijbrandij M, Winter SD, Depaoli S, Vermunt JK (2017). The GRoLTS-Checklist: Guidelines for Reporting on Latent Trajectory Studies. Structural Equation Modeling.

[CR42] van der Nest G, Lima Passos V, Candel MJJM, van Breukelen GJP (2020). An overview of mixture modelling for latent evolutions in longitudinal data: Modelling approaches, fit statistics and software. Advances in Life Course Research.

[CR43] Viner RM, Ozer EM, Denny S, Marmot M, Resnick M, Fatusi A, Currie C (2012). Adolescence and the social determinants of health. The Lancet.

[CR44] Wepf, H., Joseph, S., & Leu, A. (2021a). Benefit finding moderates the relationship between young carer experiences and mental well-being. *Psychology & Health*, 1–17. 10.1080/08870446.2021.1941961 10.1080/08870446.2021.1941961 34180332

[CR45] Wepf H, Joseph S, Leu A (2021). Pathways to Mental Well-Being in Young Carers: The Role of Benefit Finding, Coping, Helplessness, and Caring Tasks. Journal of Youth and Adolescence.

[CR46] Wong S (2017). Young carers in the NHS. British Journal of General Practice.

[CR47] Woodman, D., & Wyn, J. (2015). *Youth and generation: rethinking change and inequality in the lives of young people*. Sage.

