# Supplementary material for: Use of Parental Disability Trajectories to Identify Adolescents Who are Young Carers
Source: J Youth Adolesc. 2022 May 20;52(2):449–60. doi: 10.1007/s10964-022-01627-z (PMC9842572; doi:10.1007/s10964-022-01627-z)
Supplement: Supplementary file 1 — Supplementary Material [file 10964_2022_1627_MOESM1_ESM.docx]

**﻿SUPPLEMENTARY MATERIAL**

**Use of Parental Disability Trajectories to Identify Adolescents Who are Young Carers**

**Contents of supplementary material**

Table S1: Guidelines for Reporting on Latent Trajectory Studies (GRoLTS) checklist

Table S2: Time period between waves

Table S3: Patterns of participation and non-participation

Table S4: Missing “parental disability”

Table S5: Characteristics of sample with all missing outcome data

Table S6: Goodness-of-fit statistics for trajectory models

Table S7: Estimated polynomial coefficients from final trajectory model

Figure S2a: One class solution

Figure S2b: Two class solution

Figure S2c: Three class solution

Figure S2d: Four class solution

Figure S2e: Five class solution

Table S8: Wave 2 Sample characteristics according to each trajectory group: Imputed sample

Table S9: Odds ratios for caring by parental disability trajectory group: Complete case

**Table S1: Guidelines for Reporting on Latent Trajectory Studies (GRoLTS) checklist**

| 1 | Is the metric of time used in the statistical model reported? | Methods and Table S2 |
| --- | --- | --- |
| 2 | Is information presented about the mean and variance of time within a wave? | Table S2 |
| 3a | Is the missing data mechanism reported? | Table S3 – S5, as well as Methods section. |
| 3b | Is a description provided of what variables are related to attrition/missing data? | Tables S3 – S5 |
| 3c | Is a description provided of how missing data in the analyses were dealt with? | Methods section |
| 4 | Is information about the distribution of the observed variables included? | Methods and results sections |
| 5 | Is the software mentioned? | Methods |
| 6a | Are alternative specifications of within-class heterogeneity considered (e·g·, LGCA vs· LGMM) and clearly documented? If not, was sufficient justification provided as to eliminate certain specifications from consideration? | LGMM was considered, however we opted for LGCA because the computational demands of LGMM were not conducive to analysis with this dataset (due to sample size and time points) |
| 6b | Are alternative specifications of the between-class differences in variance–covariance matrix structure considered and clearly documented? If not, was sufficient justification provided as to eliminate certain specifications from consideration? | We used the program default, and note that our inclusion of sample weights enabled a robust estimator of the variance-covariance matrix to be calculated. |
| 7 | Are alternative shape/functional forms of the trajectories described? | Please see Table S6, Figures S2a-S2e |
| 8 | If covariates have been used, can analyses still be replicated? | N/A, covariates were not included in the model to predict trajectory groups |
| 9 | Is information reported about the number of random start values and final iterations included? | N/A |
| 10 | Are the model comparison (and selection) tools described from a statistical perspective? | Table S6, as well as Methods |
| 11 | Are the total number of fitted models reported, including a one-class solution? | Table S6 |
| 12 | Are the number of cases per class reported for each model (absolute sample size, or proportion)? | Table S6 |
| 13 | If classification of cases in a trajectory is the goal, is entropy reported? | Table S6 |
| 14a | Is a plot included with the estimated mean trajectories of the final solution? | Figure 1 in results (and Figure S2c) |
| 14b | Are plots included with the estimated mean trajectories for each model? | Figures S2a-e |
| 14c | Is a plot included of the combination of estimated means of the final model and the observed individual trajectories split out for each latent class? | No. This resulted in an incomprehensible graph. |
| 15 | Are characteristics of the final class solution numerically described (ie, means, SD/SE, n, CI, etc·)? | See Table S7 |
| 16 | Are the syntax files available (either in the appendix, supplementary materials, or from the authors)? | We describe the operational procedure of the multi-trajectory modelling in our methods section and in the Methods supplement. The syntax file for the final model is supplied by the authors on reasonable request. |

**Note**: This table presents the GRoLTS item checklist specific to trajectory studies of this kind. It notes the specific section of the manuscript or the supplementary files containing each checklist item.

**Table S2: Time period between waves**

| **Wave period** | **Mean months between waves** | **Variance of time within wave** |
| --- | --- | --- |
| 1 to 2 | 25.0 | 3.8 |
| 2 to 3 | 23.2 | 3.5 |
| 3 to 4 | 23.9 | 5.0 |
| 4 to 5 | 24.4 | 8.6 |
| 5 to 6 | 23.9 | 9.1 |
| 6 to 7 | 24.6 | 11.7 |

**Note**: This table provides the mean and variance of the number of months between each wave of data collection for the individuals included in the Longitudinal Study of Australian Children (the dataset analysed here). The mean time between waves ranged from 23.2 months (waves 2-3) to 25.0 (waves 1-2), with variance increasing across waves from 3.8 (waves 1-2) to 11.7 (waves 6-7).

﻿

**Table S3: Patterns of participation and non-participation for 4983 respondents to the Longitudinal Survey of Australian Children from Waves 1-7**

|  | N | % |
| --- | --- | --- |
| **Non-participation at wave** |  |  |
| Wave 1 | 0 | 0 |
| Wave 2 | 519 | 10.4 |
| Wave 3 | 652 | 13.1 |
| Wave 4 | 814 | 16.3 |
| Wave 5 | 1027 | 20.6 |
| Wave 6 | 1446 | 29.0 |
| Wave 7 | 1894 | 38.0 |
| **Non-participation (missing) for number of waves** |  |  |
| No wave (present all waves) | 2792 | 56.0 |
| Missing one wave | 709 | 14.2 |
| Missing two waves | 441 | 8.9 |
| Missing three waves | 271 | 5.4 |
| Missing four waves | 228 | 4.6 |
| Missing five waves | 216 | 4.3 |
| Missing six waves | 326 | 6.5 |
| Missing seven waves | 0 | 0 |
| **Patterns of non-response** |  |  |
| No wave (All complete) | 2792 | 56.0 |
| 2,3,4,5,6,7 | 326 | 6.6 |
| 3,4,5,6,7 | 179 | 3.6 |
| 2,4,5,6,7 | 18 | 0.4 |
| 2,3,5,6,7 | 12 | 0.2 |
| 2,3,4,6,7 | 4 | 0.1 |
| 2,3,4,5,7 | 2 | 0 |
| 2,3,4,5,6 | 1 | 0 |
| 4,5,6,7 | 172 | 3.5 |
| 3,5,6,7, | 17 | 0.3 |
| 3,4,6,7 | 7 | 0.1 |
| 3,4,5,7 | 1 | 0 |
| 3,4,5,6 | 1 | 0 |
| 5,6,7 | 199 | 4 |
| 4,6,7 | 23 | 0.5 |
| 4,5,7 | 6 | 0.1 |
| 4,5,6 | 0 | 0 |
| 3,6,7 | 10 | 0.2 |
| 3,5,7 | 2 | 0 |
| 3,5,6 | 0 | 0 |
| 3,4,7 | 4 | 0.1 |
| 3,4,6 | 0 | 0 |
| 3,4,5 | 0 | 0 |
| 6,7 | 317 | 6.4 |
| 5,7 | 20 | 0.4 |
| 5,6 | 12 | 0.2 |
| 4,7 | 12 | 0.2 |
| 4,6 | 7 | 0.1 |
| 4,5 | 8 | 0.2 |
| 3,7 | 11 | 0.2 |
| 3,6 | 6 | 0.1 |
| 3,5 | 1 | 0 |
| 3,4 | 3 | 0.1 |
| 7 | 484 | 9.8 |
| 6 | 89 | 1.8 |
| 5 | 27 | 0.5 |
| 4 | 28 | 0.6 |
| 3 | 26 | 0.5 |
| 2 | 55 | 1.1 |
| 0 | 2792 | 56.3 |
| 2,5,6,7 | 12 | 0.2 |
| 2,4,6,7 | 2 | 0 |
| 2,4,5,7 | 1 | 0 |
| 2,4,5,6 | 0 | 0 |
| 2,6,7 | 21 | 0.4 |
| 2,5,7 | 0 | 0 |
| 2,5,6 | 0 | 0 |
| 2,4,7 | 0 | 0 |
| 2,4,6 | 0 | 0 |
| 2,4,5 | 2 | 0 |
| 2,7 | 18 | 0.4 |
| 2,6 | 3 | 0.1 |
| 2,5 | 1 | 0 |
| 2,4 | 2 | 0 |
| 2,3,6,7 | 7 | 0.1 |
| 2,3,5,7 | 3 | 0.1 |
| 2,3,5,6 | 1 | 0 |
| 2,3,7 | 3 | 0.1 |
| 2,3,6 | 0 | 0 |
| 2,3,5 | 0 | 0 |
| 2,3 | 20 | 0.4 |
| 2,3,4,7 | 1 | 0 |
| 2,3,4,6 | 0 | 0 |
| 2,3,4 | 1 | 0 |
| 2,3,4,5 | 3 | 0.1 |

**Note**: This table provides details on the patterns of participation and non-participation for all participants in the Longitudinal Study of Australian Children. It details non-participation at specific waves, missingness for multiple waves, and non-participation for specific combinations of waves.

**Table S4: Missing “parental disability”**

| Wave | Total missing parental disability |
| --- | --- |
| 2 | 0 |
| 3 | 268 (6%) |
| 4 | 454 (10.2%) |
| 5 | 683 (15.3%) |
| 6 | 694 (15.6 %) |

**Note**: This table provides the proportion of missing parental disability information at each of the waves included in the trajectory analysis

**Table S5: Characteristics of sample with all missing outcome data**

|  |  | Missing parental disability for all waves | | Complete parental disability for at least one wave | | Chi2 (p-value) |
| --- | --- | --- | --- | --- | --- | --- |
|  |  | n | % | n | % |  |
| Gender | Male | 260 | 50.1 | 2276 | 51.0 | 0.1471, p=0.701 |
|  | Female | 259 | 49.9 | 2188 | 49.0 |  |
| Highest education of mother | Did not complete Year 12 | 155 | 30.6 | 899 | 20.3 | 49.8055, p<0.001 |
|  | Year 12 | 79 | 15.6 | 664 | 15.0 |  |
|  | Certificate/trade | 142 | 28.0 | 1152 | 26.0 |  |
|  | Diploma | 45 | 8.9 | 396 | 8.9 |  |
|  | Bachelor degree or higher | 86 | 17.0 | 1322 | 29.8 |  |
| Cultural background | Both parents born in Australia | 316 | 64.0 | 2963 | 68.4 | 6.4765, p=0.039 |
|  | 1+ parent born in English-speaking | 76 | 15.4 | 668 | 15.4 |  |
|  | 1+ parents born in non-English speaking country outside Australia | 102 | 20.7 | 703 | 16.2 |  |
| Parents in household | Two parents | 389 | 75.0 | 3897 | 87.3 | 58.9100, p<0.001 |
|  | Single parent | 130 | 25.0 | 567 | 12,7 |  |

**Note**: This table presents data on key demographic characteristics of those with missing and complete information on parental disability

| **Table S6: Goodness-of-fit statistics for trajectory models** | | | | | |
| --- | --- | --- | --- | --- | --- |
| **Number of groups** | **1** | **2** | **3** | **4** | **5** |
| BIC | -7461.82 | -6766.11 | -6754.62 | -6754.34 | -6787.30 |
| AIC | -7445.81 | -6734.09 | -6709.80 | -6703.11 | -6726.47 |
| Entropy | - | 0.771 | 0.770 | 0.637 | 0.590 |
| Mean posterior probability |  |  |  |  |  |
| Group 1 | 1.00 | 0.96 | 0.61 | 0.81 | 0.68 |
| Group 2 | - | 0.83 | 0.95 | 0.67 | 0.59 |
| Group 3 | - | - | 0.80 | 0.77 | 0.57 |
| Group 4 | - | - | - | 0.79 | 0.83 |
| Group 5 | - | - | - | - | 0.00 |
| Group membership |  |  |  |  |  |
| Group 1 | - | 84.4 | 9.7 | 62.1 | 59.4 |
| Group 2 | - | 15.6 | 80.2 | 0.6 | 2.7 |
| Group 3 | - | - | 10.0 | 28.9 | 25.6 |
| Group 4 | - | - | - | 8.4 | 0.0 |
| Group 5 |  |  |  |  | 12.4 |
|  | | | | | |

**Note**: This table presents the key goodness of fit statistics for models with 1-5 trajectory groups.

The following criteria were used to determine our chosen model:

1. Bayesian Information criterion (BIC, this provides an indication of model fit, with larger values indicating better fit)
2. Akaike Information Criterion (AIC, as for BIC, larger values are indicative of better model fit)
3. Average posterior probabilities. This provides an indication of how well individuals are classified across the trajectories, with values closer to 1.0 desirable.
4. Proportion of sample in each trajectory group (ideally this should exceed 5%).
5. Entropy. This provides an indication of the precision of class assignment. It summarises the extent to which the classes are distinct from one another on a scale of 0 to 1, with higher scores indicative of better class distinction.

The 5-group model was excluded on the basis of low group membership for two groups, and unacceptably low mean posterior probability for one group. The larger BIC and AIC values of the 3- and 4-group models indicated better model fit. While a four-group model resulted in a marginally larger BIC, it produced a group comprising of just 0.6% of the sample (at least 5% is recommended) with a lower entropy value (0.637 compared to 0.770 for the 3-group model). For this reason, the 3-group model was selected as the optimal model.

| **Table S7: Estimated polynomial coefficients from final trajectory model with three groups (n=4464)** | | | | | | |
| --- | --- | --- | --- | --- | --- | --- |
| **Polynomial term** | ***Trajectory group*** | | | | | |
|  | ***Consistently low***  ***(n=3609)*** | | ***Low-increasing-high (n=452)*** | | ***Moderate-high***  ***(n=403)*** | |
|  | **Coefficient (SE)** | **P-value** | **Coefficient (SE)** | **P-value** | **Coefficient (SE)** | **P-value** |
| **Intercept** | 9.24 (2.5) | 0.0017 | 19.91 (4.36) | <0.0001 | -173.73 (27.91) | <0.0001 |
| **Linear** | --2.95 (0.72) | <0.0001 | -7.63(1.52) | <0.0001 | 77.65 (12.17) | <0.0001 |
| **Quadratic** | 0.18 (0.04) | <0.0001 | 0.80(0.16) | <0.0001 | -12.51(1.92) | <0.0001 |
| **Cubic** |  |  | -0.03(0.01) | <0.0001 | 0.86(0.13) | <0.0001 |
| **Quartic** |  |  |  |  | -0.02(0.00) | <0.0001 |

**Note:** The table shows the estimated polynomial coefficients and their standard errors (SEs) from our final trajectory model with three groups. The P-values shown are from tests of the null hypothesis that each coefficient is equal to zero.

**Figures S2a-S2e. ﻿Predicted trajectories by group membership**


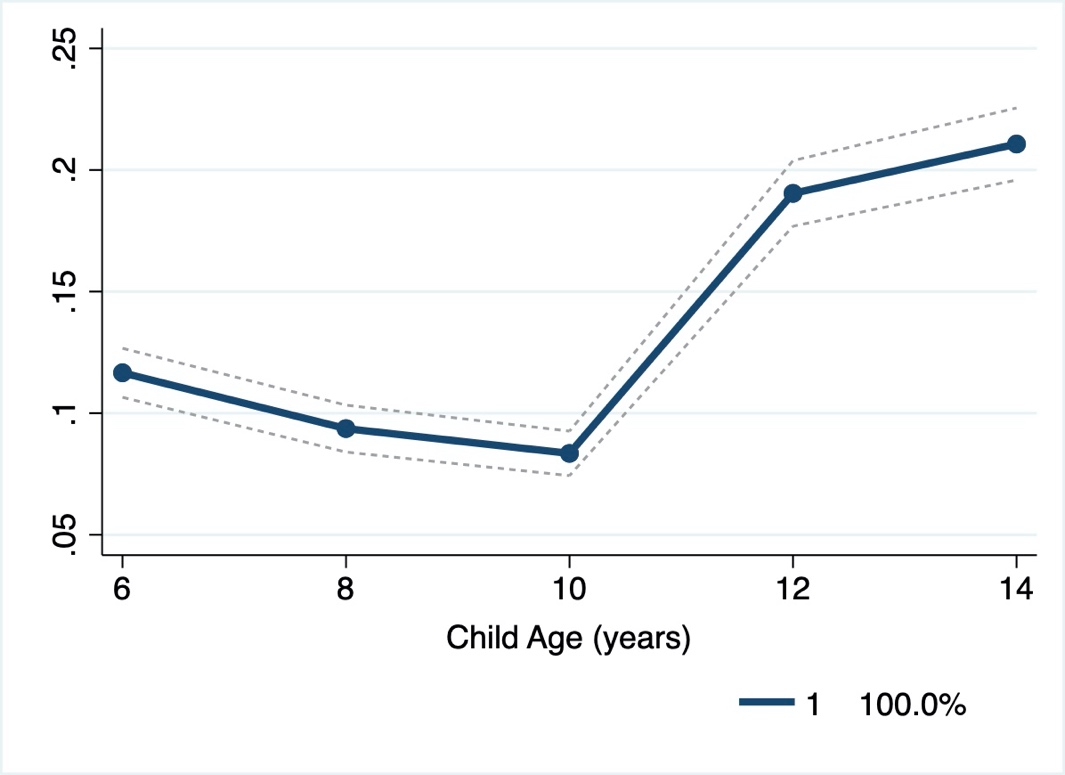


**Figure S2a: One class solution**

**Caption**: This figure plots the estimated mean trajectory for the 1-group model


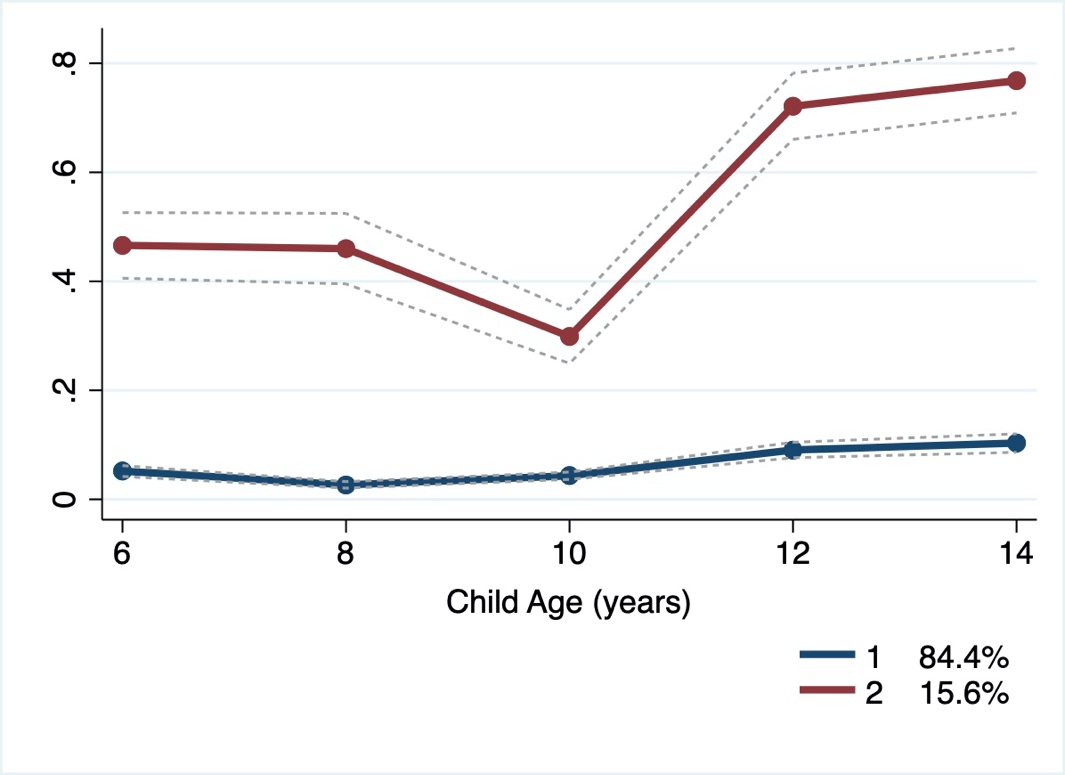


**Figure S2b: Two class solution**

**Caption**: This figure plots the estimated mean trajectories for the 2-group model


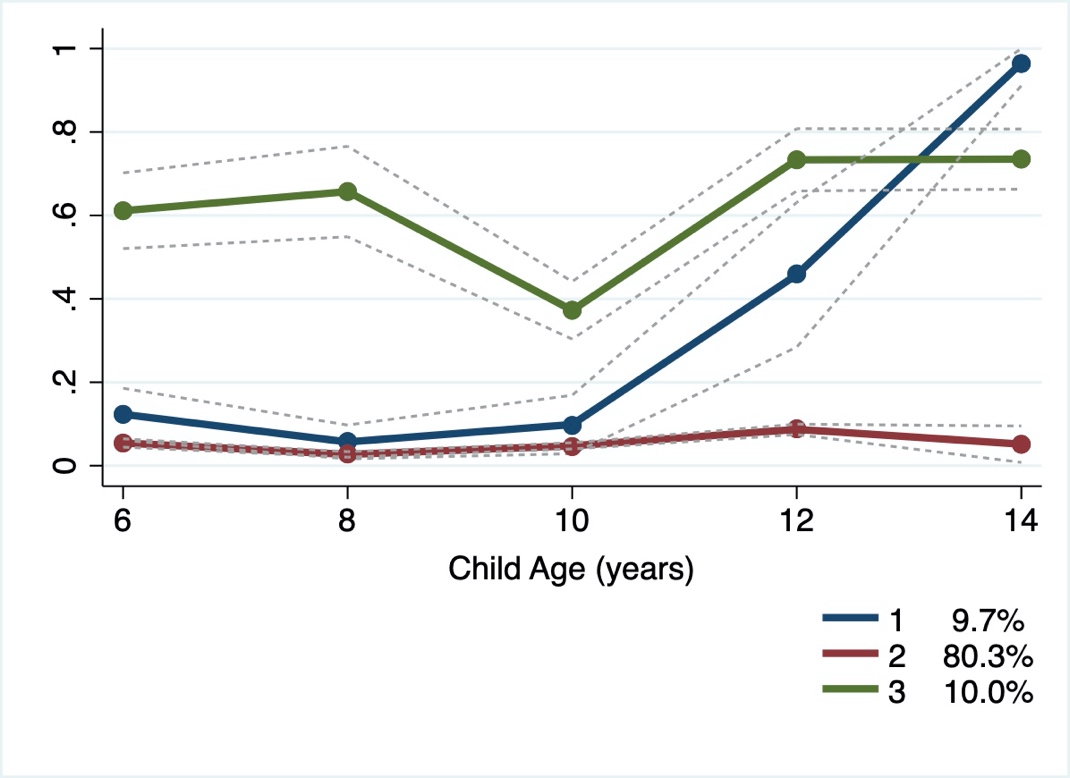


**Figure S2c: Three class solution**

**Caption**: This figure plots the estimated mean trajectories for the 3-group model


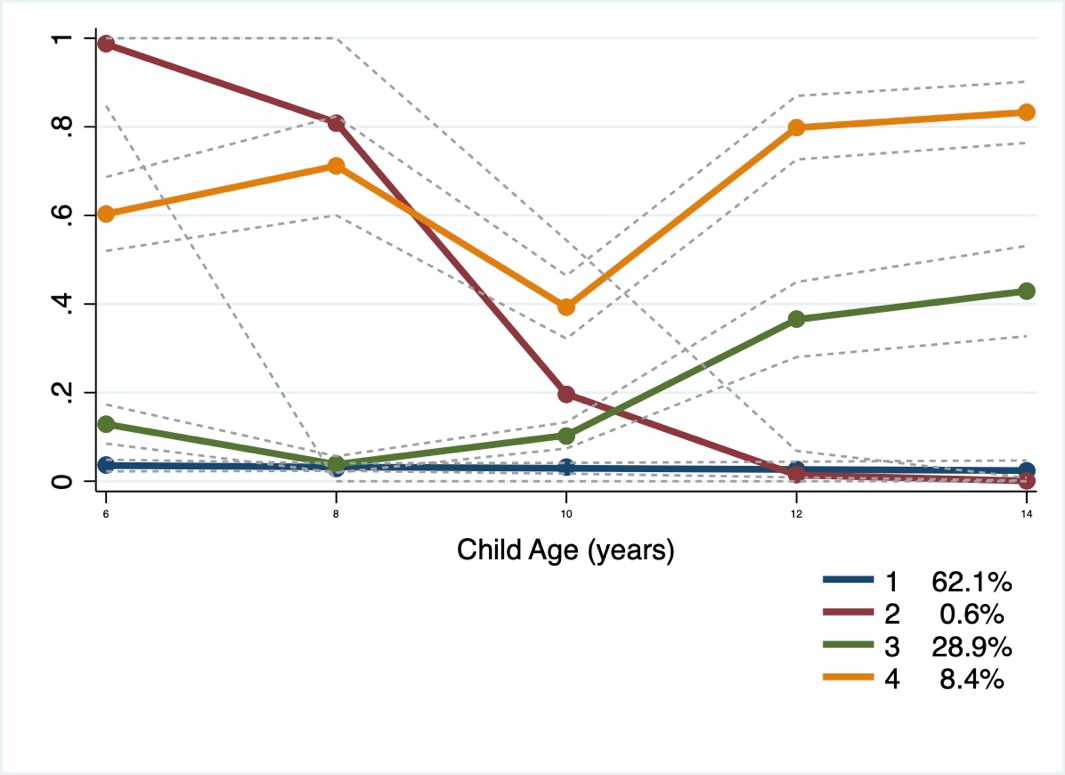


**Figure S2d: Four class solution**

**Caption**: This figure plots the estimated mean trajectories for the 44-group model


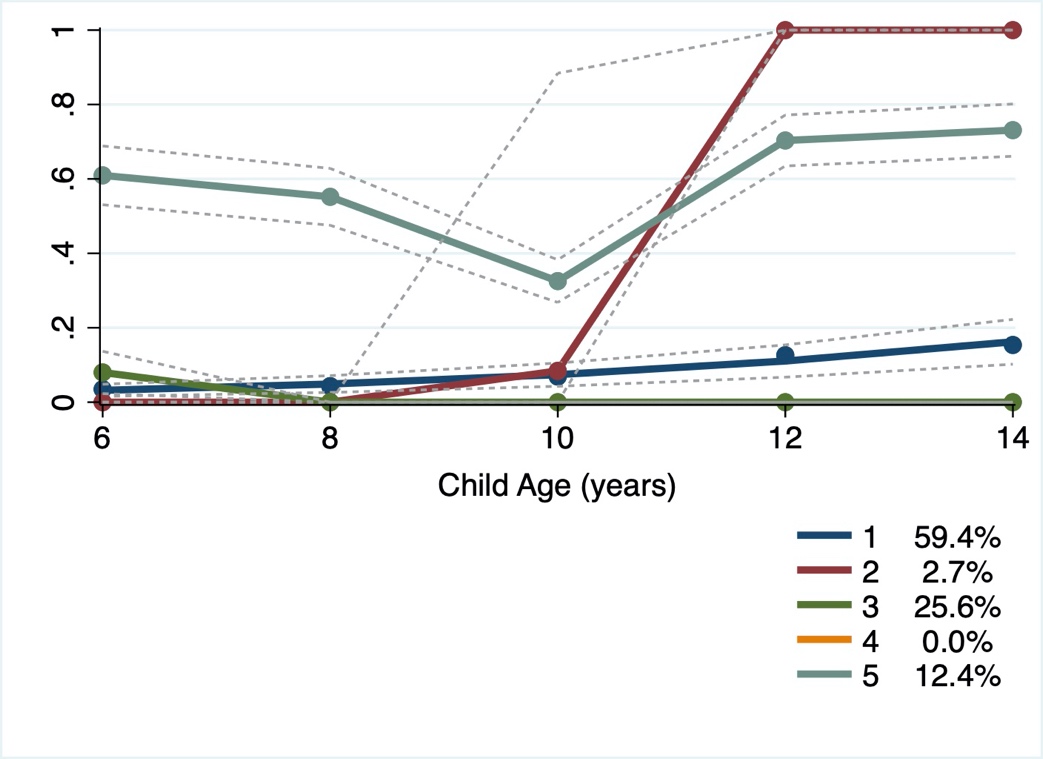


**Figure S2e: Five class solution**

**Caption**: This figure plots the estimated mean trajectories for the 5-group model

| **Table S8: Wave 2 Sample characteristics according to each trajectory group (Imputed sample: n=4464)** | | | | |
| --- | --- | --- | --- | --- |
|  |  | ***Consistently low disability***  ***n=3609*** | ***Low-increasing-high***  ***n=452*** | ***Moderate-high***  ***n=403*** |
|  |  |  |  |  |
| Gender | Male | 50.5(48.9, 52.1) | 52.0(47.4, 56.6) | 54.3(49.5, 59.2) |
|  | Female | 49.5(47.9, 51.1) | 48.0(43.4, 52.6) | 45.7(40.8, 50.5) |
| Parents in household | Two parents | 85.1(84.0, 86.3) | 86.3(83.1, 89.5) | 84.6(81.1, 88.1) |
|  | Single parent | 14.9(13.7, 16.0.) | 13.7(10.5, 16.9) | 15.4(11.9, 18.9) |
| Highest education of mother | Did not complete Year 12 | 18(16.8, 19.3) | 17.7(14.1, 21.2) | 23.9(19.7, 28.2) |
|  | Year 12 | 13.1(12.0, 14.2) | 15.3(12.0, 18.7) | 12.7(9.4, 16.0) |
|  | Certificate/trade | 29.9(28.4, 31.4) | 26.2(22.1, 30.2) | 32.5(27.9, 37.2) |
|  | Diploma | 8.8(7.8, 9.7) | 9.6(6.9, 12.4) | 8.1(5.4, 10.8) |
|  | Bachelor degree or higher | 30.2(28.7, 31.7) | 31.2(26.9, 35.5) | 22.7(18.6, 26.8) |
| Cultural background | Both parents born in Australia | 68.1(66.6, 69.7) | 70.3(66, 74.6) | 66.1(61.4, 70.8) |
|  | 1+ parent born in English-speaking | 15.0(13.8, 16.2) | 14.8(11.4, 18.1) | 19.7(15.8, 23.7) |
|  | 1+ parents born in non-English speaking country outside Australia | 16.8(15.6, 18.1) | 14.9(11.6, 18.3) | 14.1(10.7, 17.6) |
| First Nations Identity | Non-indigenous | 93.6(92.4, 94.9) | 94.6(91.9, 97.2) | 93.1(90.0, 96.3) |
|  | Indigenous | 6.4(5.1, 7.6) | 5.4(2.8, 8.1) | 6.9(3.7, 10.0) |
| Household income (quintile) | 1 - lowest | 10.6(9.5, 11.6) | 10.5(7.6, 13.4) | 17.6(13.8, 21.3) |
|  | 2 | 20.2(18.8, 21.5) | 22.2(18.3, 26.1) | 27.8(23.4, 32.2) |
|  | 3 | 32.9(31.3, 34.5) | 31.6(27.2, 36.0) | 28.9(24.4, 33.4) |
|  | 4 | 23.6(22.2, 25.0) | 22.2(18.3, 26.1) | 18.2(14.4, 22.0) |
|  | 5- highest | 12.7(11.6, 13.8) | 13.5(10.3, 16.7) | 7.6(5.0, 10.2) |
| Number of siblings in household | 0 | 8.5(7.6, 9.4) | 10.0(7.2, 12.7) | 13.9(10.5, 17.3) |
|  | 1 | 45.6(44.0, 47.3) | 43.6(39.0, 48.2) | 43.2(38.3, 48.0) |
|  | 2 | 31.0(29.5, 32.5) | 30.5(26.3, 34.8) | 26.3(22.0, 30.6) |
|  | 3 | 14.9(13.7, 16.0) | 15.9(12.6, 19.3) | 16.6(13.0, 20.3) |
| Maternal age | <30 years | 8.6(7.7, 9.5) | 6.6(4.3, 8.9) | 7.7(5.1, 10.3) |
|  | 30-34 years | 22.7(21.3, 24.1) | 25.0(21.0, 29.0) | 21.1(17.1, 25.1) |
|  | 35-39 years | 39.3(37.7, 40.9) | 35.8(31.4, 40.3) | 30.8(26.3, 35.3) |
|  | 40-44 years | 22.4(21.0, 23.7) | 22.8(18.9, 26.7) | 29.0(24.6, 33.5) |
|  | 45 years and over | 7.0(6.2, 7.8) | 9.7(7.0, 12.5) | 11.4(8.3, 14.5) |

**Note**: This table presents the characteristics of the sample at Wave 2 using the imputed sample

**Table S9: Odds ratios for caring at age 16/17 years (Wave 7) by parental disability trajectory group (complete case, n=2398)**

|  |  | ***Consistently low***  ***(n=3609)*** | ***Low-increasing-high (n=452)*** | ***Moderate-high***  ***(n=403)*** |
| --- | --- | --- | --- | --- |
| Caring (unadjusted) | | Ref | 1.54 (1.13, 2.09) | 1.51 (1.07, 2.14) |
| Caring (adjusted*) | | Ref | 1.56 (1.14, 2. 13) | 1.43 (1.00, 2.05) |

**Note**: This table presents sensitivity analyses for Table 4, and displays odds ratios for the association between parental disability trajectory and caring using the complete case sample (m=2398)

*Models adjusted for gender, parents in household, maternal education, maternal age, household income, number of siblings in household, parental country of birth, First Nations identity
